# Adding Multiple Adipokines into the Model do not Improve Weight Gain Prediction by Leptin Levels in Newborns

**DOI:** 10.4274/jcrpe.2693

**Published:** 2016-09-01

**Authors:** Consuelo Treviño-Garza, Cynthia M. Estrada-Zúñiga, Leonardo Mancillas-Adame, Laura Villarreal-Martínez, Jesús Z. Villarreal-Pérez, Isaías Rodríguez-Balderrama, Fernando F. Montes-Tapia, Manuel E. de la O. Cavazos

**Affiliations:** 1 University Autonomous of Nuevo Leon, ‘Dr. José E. González’ University Hospital, Department of Pediatrics, Monterrey/Nuevo Leon, Mexico; 2 University Autonomous of Nuevo Leon, ‘Dr. José E. González’ University Hospital, Division of Endocrinology, Monterrey/Nuevo Leon, Mexico

**Keywords:** leptin, Adipokines, prediction, weight gain, newborn

## Abstract

**Objective:**

Most adipose tissue programming is realized in early life. Also, the postnatal three months, rather than the later phases of infancy, may be more relevant in the development of an adverse cardiometabolic risk profile. The adipokines phenotype, as a predictor of early-life weight gain, has been recently explored in cord blood. To determine whether in addition to leptin levels in cord samples, adiponectin, interleukin-6 (IL-6), monocyte chemoattractant protein-1 (MCP-1), resistin, plasminogen activator inhibitor-1 (PAI-1), and tumor necrosis factor alpha (TNF-α) levels improve weight gain prediction during the first three months of life.

**Methods:**

Adiponectin, IL-6, MCP-1, leptin, resistin, PAI-1, and TNF-α were measured by multiplex immunoassay in a subsample of 86 healthy term newborns.

**Results:**

Leptin levels significantly predicted weight gain at 3 months of follow-up (r2=0.09, p=0.006). In the multivariate analysis, including additional adipokines in the model, stepwise or all at once, did not increase the prediction of weight gain after the first three months of life.

**Conclusion:**

Adding adiponectin, IL-6, MCP-1, resistin, PAI-1, and TNF-α to the prediction model of weight gain in healthy newborns did not prove to be useful. It is probable that their relative contribution to weight gain is not important. Only leptin was relevant as a predictor of weight gain at the 3-month endpoint.

**WHAT IS ALREADY KNOWN ON THIS TOPIC?**

The adipokines phenotype, as a predictor of early-life weight gain, has been recently explored in cord blood serum concentrations. Leptin levels significantly predicted weight gain at 3 months of follow-up.

**WHAT THIS STUDY ADDS?**

Adding adiponectin, interleukin-6, monocyte chemoattractant protein-1, resistin, plasminogen activator inhibitor-1, and tumor necrosis factor alpha to the prediction model of weight gain in healthy newborns was not useful.

## INTRODUCTION

Most of adipose tissue programming and therefore, adulthood obesity, has its origin in early stages of life; i.e. during intrauterine life and the breastfeeding period (1,2,3). Accelerated growth during the first 24 months of life has been associated with an adverse cardiometabolic profile and metabolic syndrome (4). Within these first two years of life, the initial three months may be more relevant in the development of an adverse cardiometabolic risk profile (5,6).

The adipokines phenotype as predictor of early-life weight gain, has been recently explored, both in breast milk and cord blood serum concentrations (7,8,9). Adiponectin and leptin are factors known to influence normal and abnormal fetal growth (10,11,12). Low adiponectin concentrations have been reported in adults and in infants who were born small for gestational age, linking growth restriction with a cardiometabolic profile later in life (13).

Other factors which have been identified in breast milk are normally secreted in adipocytes [interleukin-6 (IL-6); monocyte chemoattractant protein-1 (MCP-1), resistin, tumor necrosis factor-α (TNF-α)] (14). These additional factors in breast milk, have not yet been studied in cord blood as predictors of weight gain during the early postnatal period.

The aim of this study was to determine if, in addition to the leptin concentration in a cord sample, the cord blood levels of adiponectin, IL-6, MCP-1, resistin, plasminogen activator inhibitor-1 (PAI-1), and TNF-α improve the prediction of weight gain during the first three months of life in a cohort of healthy term newborn subjects.

## METHODS

A subsample of a previously reported cohort (3) of healthy normal weight at-term newborns is reported in this study. The original study population was composed of 99 infants born at the University Hospital of the Universidad Autónoma de Nuevo Leon in Monterrey, Mexico between January 2006 and December 2007. The subset is a convenience sample of those subjects from the original cohort whose blood cord serum of a sufficient quantity (25 µL) had been stored to run a multiplex immunoanalysis (MIA). Also, both baseline and at age 3 months (± one week) weight data were available for these subjects.

Weight was measured at birth, in duplicate, using a Torrey calibrated scale (Torrey, S.A. de C.V., Monterrey, Mexico) in the delivery room and during the 3-month follow-up visit at the outpatient clinic.

The cord blood samples were obtained from the umbilical cord vein immediately after birth and centrifuged at 3000 rpm. Serum was separated and stored at -20 °C in aliquots for later analysis. Samples were thawed only once at the time of the analysis.

The study was approved by the Ethics and Research Committee of the ‘Dr. José Eleuterio González University Hospital of the Universidad Autónoma de Nuevo León. Parents gave written informed consent before data collection and blood sampling.

Adiponectin, IL-6, MCP-1, leptin, resistin, PAI-1, and TNF-α were measured by MIA using an ad doc human serum adipokine kit (96-well plate assay Cat# HADK2-61K-B, Millipore Corp, St. Charles, MO) on a Luminex TM 200 analyzer system (Luminex Corporation, Austin, TX). Sensitivity and intra-/inter-assay variation coefficients are shown in Table 1.

### Statistical Analysis

Central tendency measures are expressed as mean ± standard deviation values unless otherwise specified. Those variables with a non-normal distribution were transformed to Ln for analysis. A stepwise linear regression analysis was performed to find variables in addition to leptin which may increase the accuracy of the weight-gain-at-month-3 prediction equation. A p-value ≤0.05 was considered statistically significant. All analyses were performed using IBM SPSS Statistics for Mac v.21.0 (Chicago, IL, USA).

## RESULTS

The study population included 86 term newborns (male n=41, 47.7%). The feeding type was available in 85 of the subjects (breastfeeding, n=43; formula-feeding n=42). Mean gestational age was 39.24±1.2 weeks. Mean birthweight was 3.31±0.45 kg. On average, the weight gain at age 3 months was 6.19±0.72 kg and 88.94% of the baseline weight (2.87±0.63 kg) (Table 2). No signs of morbidity were encountered in the study sample during the 3-month follow-up period.

Adiponectin, IL-6, MCP-1, leptin, and resistin were transformed to Ln for analysis. TNF-α and PAI-1 had a normal distribution and did not require transformation. Baseline Ln adiponectin was 3.79±0.94 μg/mL; Ln IL-6 was 1.99±1.27 pg/mL; Ln MCP-1 was 5.92±0.53 pg/mL; Ln leptin levels were 3.53±0.91 ng/mL; Ln resistin was 4.1±1.4 ng/mL; TNF-α was 52.29±4.63 pg/mL; and PAI-1 was 53.37±16.3 ng/ mL (Table 3).

Leptin levels weakly but significantly predicted weight gain at 3 months for the whole population (r2=0.09, p=0.006). In the multivariate analysis, including either a stepwise or an all at once approach, the additional adipokines available to the model did not increase the prediction of weight gain after the 3-month follow-up period. When analysing the population by feeding type during the 3-month period, no differences were found vs. the full cohort analysis.

## DISCUSSION

It has been demonstrated that rapid weight gain in both small-for-gestational age and appropriate-for-gestational age full-term infants during the first 3 months of life is associated with insulin resistance and hypertriglyceridemia in adult life (6). In a twins study, the relative influence of both environmental and intrinsic subject characteristics has been analysed, and both components were reported to have an association with cardiovascular risk factors in adult life (5). In a previous analysis, we reported the association of cord blood leptin levels and type of feeding with weight gain at 3 months of life (15). In recent years, the technology for cytokine measurement has evolved and became more accessible. Also, a lower sample volume is required to conduct multiple analyses simultaneously (16). We therefore foresaw an opportunity to explore whether adding other cytokines to the prediction model would increase its efficacy or not.

Other approaches to evaluate the prediction of weight gain in different time windows have been reported. Nakano et al (17) found that adiponectin levels in cord serum significantly predict body mass index (BMI) z-score gains from birth to 3 years of age in Japanese infants; however, when only the first 6 months were analysed, it was found that adiponectin levels do not predict postnatal BMI z-score gains, a finding that suggests that energy intake influenced by breast feeding and leptin levels may be stronger factors that affect changes in BMI z-scores than adiponectin levels during this period. Meanwhile, Mazaki-Tovi et al (13) reported that adiponectin concentrations negatively correlate with weight and BMI at one year of age, and that leptin concentrations positively correlate with weight and size at one year, suggesting the role of both adipokines in postnatal growth. Mantzoros et al (9) concluded that low leptin levels in cord blood are associated with pronounced weight gain in the first six months of life and with a higher BMI at 3 years of age, while adiponectin levels in cord blood are inversely associated with weight gain in the first 6 months of life and predict an increase in central adiposity at 3 years of age. These findings highlight the fact that the prenatal and early postnatal period are of developmental plasticity. It can be speculated that during such critical periods, long-term metabolic pathways that become relatively resistant to change are activated.

The association between adiponectin and weight at one month of age was not demonstrated by Inami et al (18), suggesting that adiponectin has an effect on fetal growth but no effect on early postnatal growth, at least in the first month of life.

It is important to point out the large diversity in the methodologies as well as in the study populations, and time periods of these studies could explain the inconsistency of the results.

In our study, the variance of weight gain explained by leptin was 9%, while the addition of adiponectin, IL-6, MCP-1, resistin, PAI-1, and TNF-α to the model explained a variance of 11.7%, this latter finding remaining significant only by determining leptin as a predictor. Thus, the addition of these adipokines to the model in our study did not prove useful in predicting weight gain in three-month term newborns born with appropriate weight for gestational age. However, in subjects at the extremes of birth weight or in other time periods, these molecules may play a role in explaining weight gain in our study group, subjects that were essentially healthy, but it is unlikely that their relative contribution to weight gain is important.

Given that in our study population, both feeding types (namely breastfeeding and formula-feeding) are similarly represented, that both genders are equally distributed, and that the study population was intentionally selected as a healthy term-newborn population with normal weights for gestational age and that epidemiologic, nutritional and stress-related factors were not perceived to bias the study outcome, it is fair to state that among the cytokines studied, only leptin was relevant as a predictor of weight gain at the 3-month endpoint. Although this was a negative study with regard to the added value of other cytokines to the model, it is an important contribution to the understanding of weight change in the early developmental period.

## Ethics

Ethics Committee Approval: The study was approved by the Ethics and Research Committee of the ‘Dr. José Eleuterio González University Hospital of the Universidad Autónoma de Nuevo León, Informed Consent: Parents gave written informed consent before data collection and blood sampling.

Peer-review: Externally peer-reviewed.

## Figures and Tables

**Table 1:**
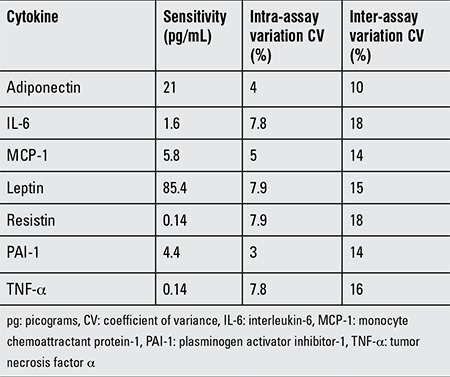
Sensitivity and intra-/inter-assay coefficients of variation

**Table 2:**
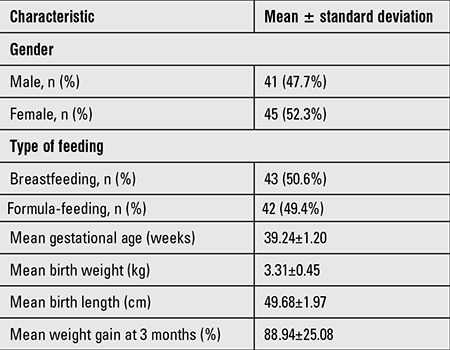
Neonatal characteristics of the study group (n=86)

**Table 3:**
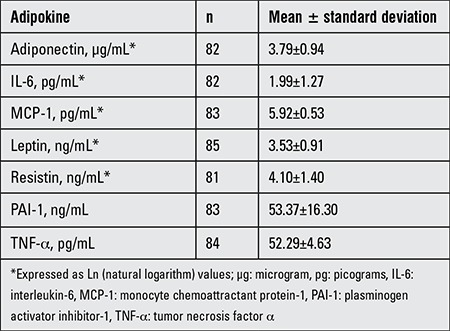
Adiponectin, interleukin-6, monocyte chemoattractant protein-1, leptin, resistin, plasminogen activator inhibitor-1, and tumor necrosis factor α concentrations in the umbilical cord blood of the subjects
